# Protonation Modification of Commercial OPBI as Binders Toward High‐Performance High‐Temperature PEM Fuel Cells

**DOI:** 10.1002/advs.78090

**Published:** 2026-09-28

**Authors:** Jinzhen Huang, Minhui Liang, Jingjing Lin, Zhihuan Pan, Jinwu Peng, Lei Wang

**Affiliations:** ^1^ Guangdong Provincial Key Laboratory of New Energy Materials Service Safety Shenzhen Key Laboratory of Polymer Science and Technology College of Materials Science and Engineering Shenzhen University Shenzhen People's Republic of China; ^2^ Zhangjiang Laboratory Pudong Shanghai People's Republic of China; ^3^ Key laboratory of Resources Environmental and Green Low Carbon Processes in East Guangdong School of Materials Science and Engineering Hanshan Normal University Chaozhou People's Republic of China

**Keywords:** catalyst layers, high‐temperature proton exchange membrane fuel cells, ionize polybenzimidazole binders, protonated polybenzimidazole, triple‐phase boundaries

## Abstract

Polybenzimidazole (PBI) is a promising binder for catalyst‐layer (CL) of high‐temperature proton exchange membrane fuel cells (HT‐PEMFCs), offering acid‐doped proton conductivity and better membrane compatibility than PTFE. However, commercial PBI binders suffer from chain aggregation and nonuniform distribution, which block Pt active sites and compromise fuel cell performance. Herein, we demonstrate a facile formic‐acid protonation strategy to regulate commercial OPBI binders without disrupting the polymer backbone. Protonation introduces positively charged benzimidazole sites, inducing interchain electrostatic repulsion that loosens chain packing, enlarges intermolecular spacing, and promotes uniform binder dispersion over catalyst surfaces. As a result, protonated OPBI exhibits a 382% increase in phosphoric acid uptake and a proton conductivity of 357 mS cm^−1^. In CL, protonated OPBI reduces proton transport resistance by 63.7%, thereby promoting triple‐phase boundary formation, as evidenced by nearly doubled ECSA, while preserving reactant‐transport pathways. The resulting membrane electrode assembly delivers a peak power density of 912 mW cm^−2^ and a Pt‐mass‐specific peak power density of 5.2 W mg_Pt_^−1^ at an ultralow Pt loading of 0.075 mg cm^−2^. This work highlights protonation‐induced chain‐packing regulation as an effective strategy for high‐performance PBI binders in HT‐PEMFCs.

## Graphical abstract

Commercial OPBI binders is regulated via a facile formic‐acid protonation strategy without disrupting the polymer backbone. Protonation introduces positively charged benzimidazole sites, inducing interchain electrostatic repulsion that loosens chain packing, enlarges intermolecular spacing, and promotes uniform binder dispersion over catalyst surfaces.

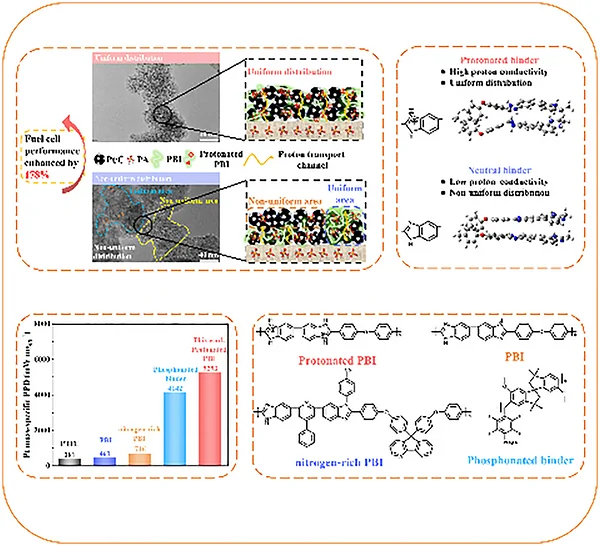

## 1 Introduction

Proton exchange membrane fuel cells (PEMFCs) are regarded as a key technology for sustainable energy conversion because of their high power density and zero‐emission operation [1, 2, 3]. Compared with conventional low‐temperature PEMFCs, high‐temperature PEMFCs (HT‐PEMFCs), typically operating at 120–200°C, offer several advantages, including faster oxygen reduction kinetics, enhanced CO tolerance, and simplified thermal and water management. In particular, reducing Pt loading can lower noble‐metal catalyst costs, while maintaining high mass‐specific power is advantageous for lightweight transportation applications, where compact and weight‐efficient power systems are highly desirable [4, 5, 6, 7]. Within the membrane electrode assembly (MEA), the catalyst layer (CL) serves as the primary electrochemical reaction zone, and its composition and microstructure critically determine cell performance and Pt utilization [8, 9, 10]. In low‐temperature PEMFCs, perfluorosulfonic acid ionomers such as Nafion function effectively as both proton‐conducting ionomers and binders under hydrated conditions. However, their reliance on water‐assisted proton transport results in a dramatic loss of conductivity above 100°C, rendering them unsuitable for HT‐PEMFC operation. In contrast, commercial CLs commonly use polytetrafluoroethylene (PTFE) as the binder. However, the intrinsically nonconductive PTFE provides no proton‐transport capability and shows poor interfacial compatibility with polybenzimidazole (PBI)‐based membranes, leading to a high interfacial resistance of 9.48 Ω cm^2^, a low peak power density of 381.2 mW cm^−2^, and a limited electrochemically active surface area (ECSA) of 14.83 cm^2^ mg_Pt_^−1^ [11, 12, 13]. Accordingly, developing high‐performance CL binders that simultaneously enable efficient proton transport, strong catalyst binding, and low‐resistance interfacial contact remains a central challenge for HT‐PEMFCs. Aromatic PBI‐based polymers containing benzimidazole heterocycles have emerged as promising binder candidates for HT‐PEMFC CLs because of their excellent thermal and chemical stability and strong affinity for phosphoric acid (PA) [14, 15]. Proton conduction in PBI is achieved via PA doping, where the basic nitrogen sites of the benzimidazole units promote acid uptake and facilitate proton hopping under anhydrous high‐temperature conditions [16, 17, 18, 19]. Despite these advantages, state‐of‐the‐art PBI binders still suffer from insufficient proton conductivity and excessive coverage of catalyst active sites, leading to limited ECSA, poor Pt utilization, and unsatisfactory power output. For example, a representative meta‐PBI binder exhibits a PA uptake of 425% and a proton conductivity of 30–60 mS cm^−1^; nevertheless, the resulting CL delivers an ECSA of only 21 cm^2^ mg_Pt_^−1^, a Pt utilization of merely 2.5%, and a peak power density of 325 mW cm^−2^ at 160°C [7, 20] These metrics remain far below the performance levels required for practical low‐Pt HT‐PEMFC applications.

Considerable efforts have therefore been devoted to developing advanced binders to improve proton conduction and Pt utilization in CLs. Representative strategies include phosphonated ionomeric binders [21, 22, 23], nitrogen‐rich PBI derivatives [4, 24, 25], and other chemically modified polymers with tailored proton‐conducting functionalities [12]. For example, Li et al. [22] reported a phosphonated ionomeric binder (PFPA‐PIM‐SBI) that achieved a Pt mass‐specific peak power density of 4.2 W mg_Pt_^−1^ at 160 °C. Gao et al. [25]. developed a rigid nitrogen‐rich PBI derivative containing pyridine, diazofluorene, and nitrile moieties, enabling stepwise protonation and hydrogen‐bond‐assisted proton transport and delivering 0.716 W mg_Pt_^−1^. Such strategies enhance proton conduction by introducing additional basic sites but generally require chemical modification of the PBI backbone. Despite these advances, most reported PBI‐based binders still deliver moderate Pt‐mass‐specific power densities of only ≈0.7–1.4 W mg_Pt_^−1^ and often rely on multistep syntheses or costly monomers. A simpler, structure‐preserving strategy is therefore highly desirable. Beyond molecular‐structure design, the protonation state of PBI binders remains largely underexplored, despite its potential influence on chain packing, PA affinity, proton transport, and catalyst accessibility in HT‐PEMFC catalyst layers.

Here, we report a simple protonation‐state engineering strategy for commercial OPBI binders in HT‐PEMFC catalyst layers. FA provides sufficient acidity to protonate benzimidazole sites while its high volatility enables facile removal, allowing the OPBI protonation state to be regulated without chemical modification of the polymer backbone (Scheme 1). Although ionic binders can perturb colloidal interactions in catalyst inks, formic‐acid protonation preserves homogeneous binder dispersion. Protonated, neutral, and deprotonated OPBI binders were obtained by solution treatments, enabling the role of protonation state to be examined without changing the polymer backbone. Protonation modulates interchain packing, enhances PA uptake, and improves binder distribution and catalyst accessibility, thereby facilitating proton transport and Pt utilization. Benefiting from these effects, the optimized MEA achieves a Pt‐mass‐specific peak power density of 5.25 W mg_Pt_^−1^ at 160°C with an ultralow cathode Pt loading of 0.075 mg cm^−2^, among the highest values reported for PBI‐binder‐based HT‐PEMFCs. This work provides a facile route to high‐performance PBI‐based binders for low‐Pt HT‐PEMFCs.

**SCHEME 1:**
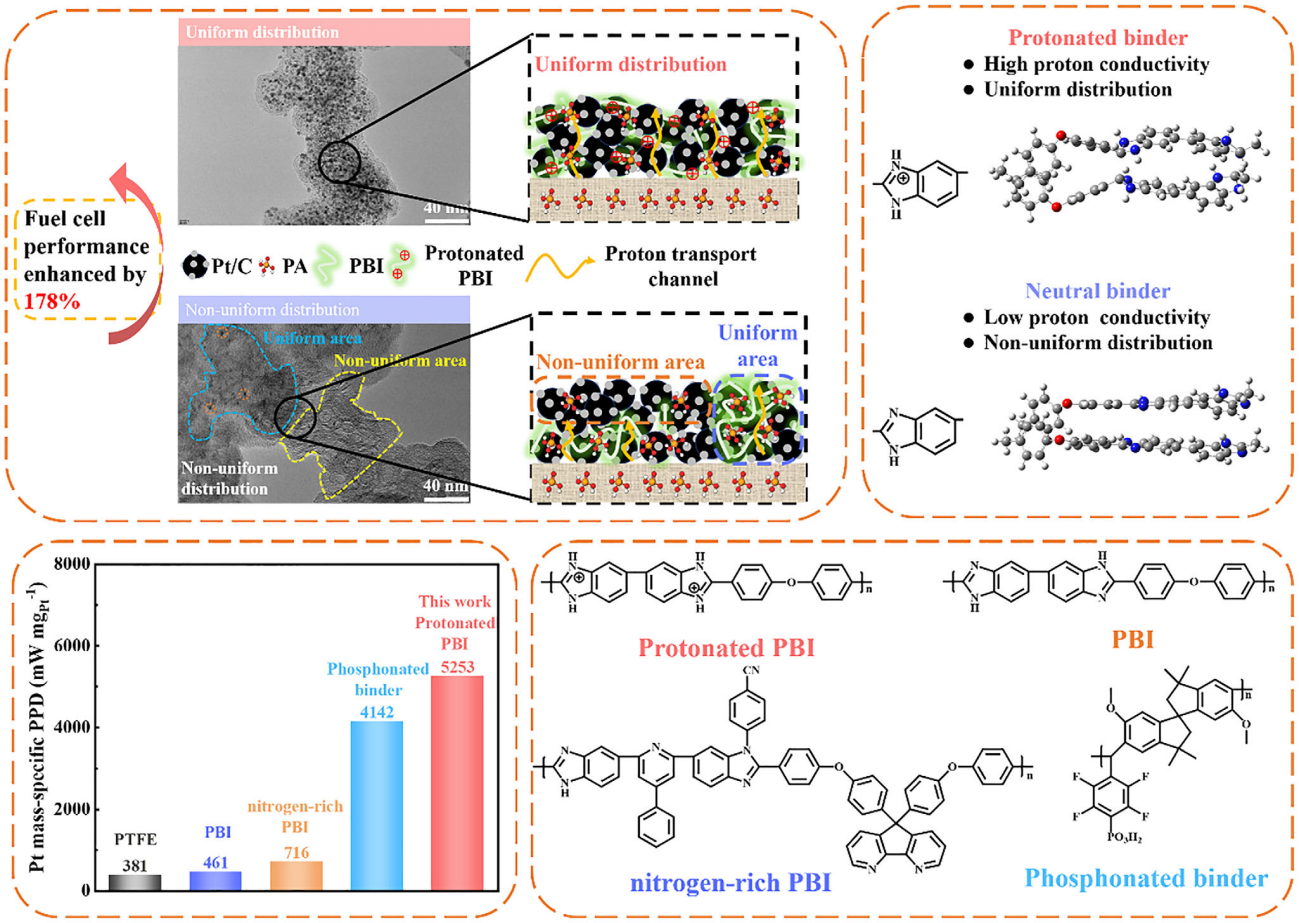
Concept of optimizing fuel cell performance through ionized PBI binders.

## 2 Result and Discussion

### 2.1 Structure Characterization and Mechanism of Ionized OPBI

The synthesis process of OPBI and mPBI are shown in Figure S1a,b. Figure S1c shows the ^1^H NMR spectra of OPBI and mPBI. The signal at 13.00 ppm in the OPBI spectrum corresponds to the imidazole ring hydrogen, whereas the signal at 13.50 ppm in the mPBI spectrum corresponds to the analogous hydrogen. These distinct chemical shifts confirm the successful synthesis of both polymers. Furthermore, characteristic signals observed at 6.60 and 7.01 ppm indicate that the copolymer was end‐capped by 3,3′‐diaminobenzidine during polymerization [17, 26]. Additionally, Figure S1d shows the FT‐IR spectrum exhibits bands at 3427 and 3053 cm^−1^, corresponding to N–H stretching vibrations in the imidazole rings. The band at 1594 cm^−1^ is attributed to C = N stretching vibrations, while the band at 1291 cm^−1^ arises from C–N stretching vibrations within the imidazole rings. A band at 1233 cm^−1^ corresponds to the vibration of the ether linkage (C–O–C) in the main chain [14]. These spectral features further confirm the successful synthesis of the polymers.

To elucidate how different protonation states affect OPBI conformation, we analyzed their π–π stacking configurations and calculated the corresponding intermolecular binding energies and geometric parameters. Further insight was obtained from three model compounds representing protonated OPBI (POPBI), neutral OPBI (NOPBI) and deprotonated OPBI, (DOPBI). As shown in Figure 1a, multiple weak noncovalent interactions exist within the stacked OPBI structures, and the benzimidazole moieties adopt a slipped π–π stacking arrangement dominated by van der Waals interactions. Among the three states, POPBI exhibits the least negative binding energy (−76.36 kJ mol^−1^), compared with NOPBI (−143.13 kJ mol^−1^) and DOPBI (−102.40 kJ mol^−1^), indicating the weakest interchain association and the least stable stacked configuration. This reduced binding strength is attributed to protonation of the benzimidazole units, which weakens favorable intermolecular interactions and introduces electrostatic repulsion between positively charged sites. As a result, the polymer chains become less compactly packed, with reduced interchain interpenetration and increased spatial separation. Accordingly, upon protonation, the centroid‐to‐centroid distance increases from 3.83 Å in NOPBI to 4.37 Å in POPBI, while the displacement angle increases from 24.29° to 26.56° (Figure 1b,c). These results indicate that protonation weakens and distorts the π–π stacking arrangement, leading to a more extended and loosely packed chain configuration.

**FIGURE 1:**
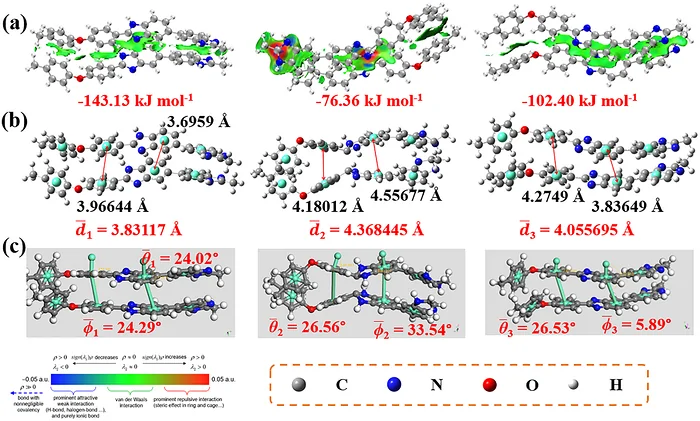
π–π stacking of the OPBI in different states. (a) The IGMH images of the OPBI structural unit and the interaction energy of the OPBI calculated at the structural unit's level; (b) A centroid‐to‐centroid distance (*d*) of OPBI; (c) Displaced angle (*θ*) and twist angle (*ϕ*) of OPBI. From left to right, they are neutral OPBI, protonated OPBI and deprotonated OPBI.

### 2.2 The Intrinsic Performance of Ionized OPBI

OPBI was treated in different media to establish distinct protonation states and to clarify the accompanying chemical and structural changes [27, 28]. As shown in Figure S2, thermogravimetric analysis reveals mass losses of ≈5% for NOPBI and ≈8% for POPBI in the range of 200–300°C, indicating the presence of residual treatment media. This behavior is consistent with the difficulty of completely removing high‐boiling‐point NMP during drying, as well as the strong acid–base association between FA and benzimidazole units in PBI [29]. Such residual species may influence the subsequent thermal and spectroscopic characteristics of the treated polymers. X‐ray photoelectron spectroscopy (XPS) further elucidates the nitrogen chemical states in the differently treated OPBI samples (Figure 2a). In POPBI, three N 1s components centered at ≈398.27, 399.98, and 400.69 eV are assigned to ═N─, ─NH─, and protonated ═NH^+^─ species (0.28:1:0.55), respectively, confirming successful protonation (33.7%) [30]. In contrast, DOPBI exhibits three N 1s peaks at ≈398.23, 399.62, and 400.27 eV, corresponding to deprotonated nitrogen species, ═N─, and ─NH─ groups (0.31:0.52:1), consistent with successful deprotonation (37.3%) [31]. NOPBI, by comparison, displays only two N 1s peaks at ≈398.27 and 400.01 eV (0.34:1), characteristic of pure PBI (0.88:1). Additional spectroscopic evidence supports the strong interaction between FA and OPBI. As shown in Figure S3, the FT‐IR spectra display a band at 1637 cm^−1^ attributable to the C = O stretching vibration of residual FA or NMP, while the N–H stretching band at 3053 cm^−1^ becomes weaker after FA treatment. ^1^H NMR analysis (Figure 2b) further shows that FA persists in OPBI after various thermal treatments and can be completely removed only after alkaline soaking in 1 M NaHCO_3_ for 24 h, indicating strong association between FA and the polymer. Compared with pristine OPBI, FA‐treated OPBI exhibits pronounced changes in the benzimidazole proton region; notably, the original N–H resonance at ≈13 ppm disappears after protonation, consistent with previous reports on protonated PBI systems and indicative of substantial changes in the local electronic environment induced by FA treatment [19, 30].

**FIGURE 2:**
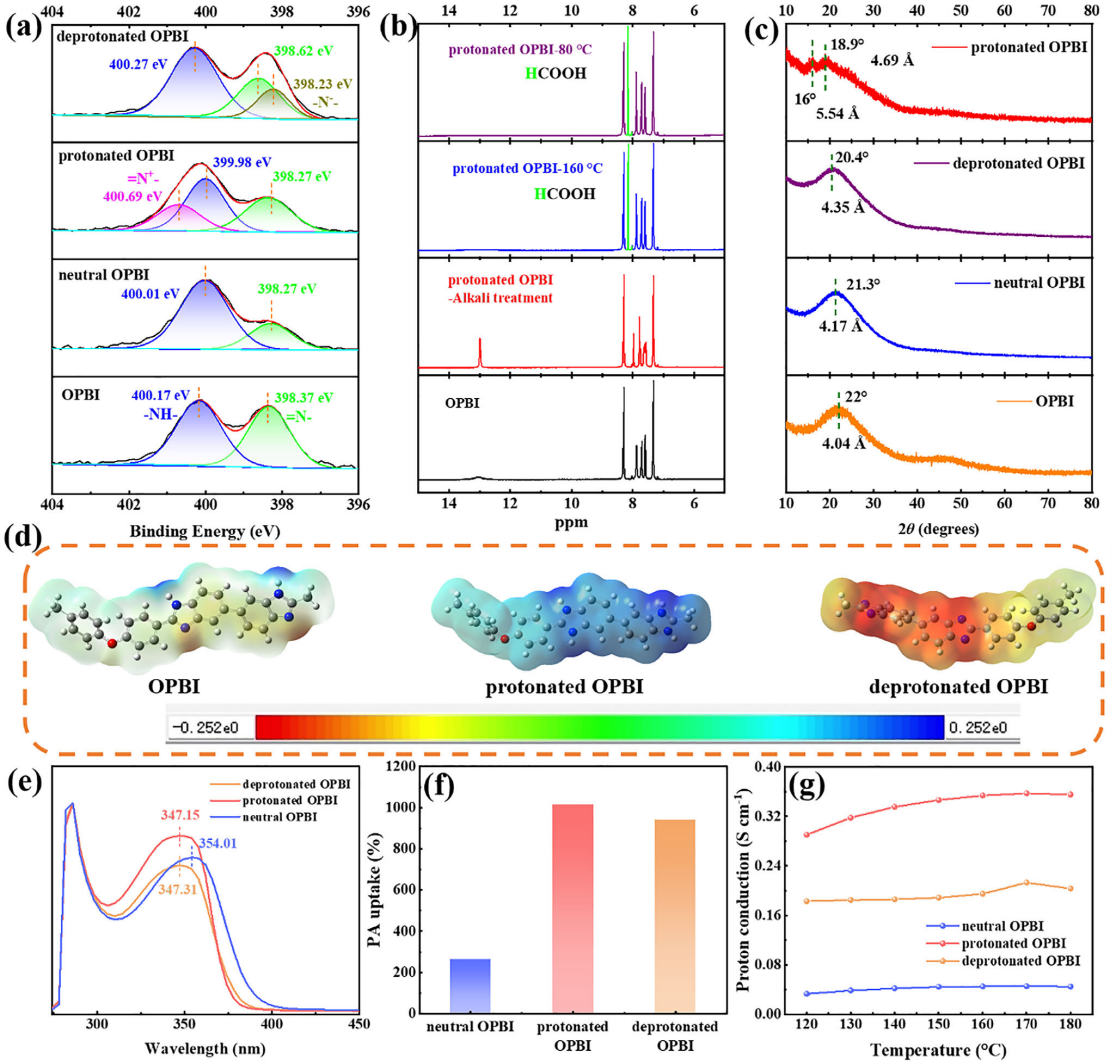
Structural and functional properties of OPBI in different states. (a) XPS N 1s spectra of OPBI in different states; (b) ^1^H NMR spectra of POPBI under various treatments; (c) XRD patterns confirming amorphous structure of OPBI; (d) Electrostatic potential (ESP) distribution maps of OPBI. Red regions indicate negative electrostatic potentials (electron‐rich areas), while blue regions represent positive potentials (electron‐deficient areas); (e) The ultraviolet‐visible absorption spectrum of OPBI; (f) PA uptake of OPBI; (g) Temperature‐dependent proton conductivity.

Structural characterization further reveals that the protonation state strongly affects chain packing. As shown in Figure 2c, all OPBI samples remain essentially amorphous, as evidenced by their broad XRD features. The corresponding d‐spacings fall in the range of 4.04–5.54 Å, with DOPBI and POPBI exhibiting larger interchain spacings than NOPBI, in agreement with the simulation results. FA protonates the benzimidazole units and introduces cationic sites along the polymer chains, whereas NaOH treatment generates deprotonated nitrogen sites and weakens the original interchain hydrogen‐bonding interactions. In both cases, the altered charge state disrupts compact chain packing and increases chain‐to‐chain separation, thereby creating more free volume for PA accommodation (Figure S4). The N_2_ adsorption results at 77.3 K support this trend: DOPBI shows the highest BET surface area (0.488 m^2^ g^−1^) and total pore volume (1.402 cm^3^ kg^−1^ at P/P_0_ = 0.99), followed by POPBI (0.1772 m^2^ g^−1^ and 0.485 cm^3^ kg^−1^) and NOPBI (0.0938 m^2^ g^−1^ and 0.061 cm^3^ kg^−1^). These results collectively indicate that tuning the protonation state modulates the packing density and accessible free volume of OPBI, which is expected to strongly influence subsequent PA uptake and proton transport.

In CLs, PA serves as the primary proton‐conducting medium. However, the tightly packed chains of neutral OPBI limit PA uptake and therefore restrict proton transport within the binder phase. Enhancing the acid‐hosting capability of the binder through protonation‐state regulation is thus of central importance. To clarify how different OPBI states influence phosphate accommodation, the electrostatic potential (ESP) distributions were analyzed. As shown in Figure 2d, the three OPBI states exhibit markedly different surface charge distributions, with red and blue regions representing electron‐rich and electron‐deficient areas, respectively. In POPBI, protonation generates positively polarized domains that are favorable for phosphate uptake and for the formation of continuous acid‐mediated proton‐transport pathways [21]. Consistent with this interpretation, the UV–vis spectra of charged OPBI states exhibit a blue shift relative to NOPBI (Figure 2e), indicating weakened electronic conjugation and reduced intermolecular π–π coupling after protonation/deprotonation [32, 33]. This spectral change agrees well with the enlarged interchain spacing revealed by XRD (Figure 2c), suggesting a less compact packing structure that is more favorable for acid accommodation.

To evaluate the intrinsic acid‐hosting and proton‐conduction capability of the binder phase, the differently treated OPBI samples were immersed in PA for 24 h before measuring PA uptake and anhydrous proton conductivity. As shown in Figure 2f,g, POPBI exhibits the highest PA uptake (1016%) and proton conductivity (357 mS cm^−1^ at 160°C), far exceeding those of NOPBI (45 mS cm^−1^) and DOPBI (194 mS cm^−1^). FA can protonate the nitrogen atoms in imidazole groups during polymer solvation, and the positively charged nitrogen atoms prevent the formation of intermolecular hydrogen bonds among polymers by electrostatic repulsion [30]. Hence, the free volume and amorphous state of the POPBI were superior to those of NOPBI. This remarkable enhancement is attributed to the protonation‐induced loosening of chain packing, the substantially increased PA uptake, and the resulting formation of more efficient proton‐transport pathways within the binder phase [34, 35]. Compared with previously reported proton conductivities of 50.6–170 mS cm^−1^ for PA‐doped PBI‐based materials, this value represents one of the highest conductivities reported to date [16, 29, 36, 37]. Contact‐angle measurements further support the superior PA wettability of POPBI (Figure S5). POPBI exhibits the lowest contact angle (21.73°), compared with NOPBI (25.30°), DOPBI (53.42°) and commercial PTFE (126.29°), indicating the strongest affinity toward PA wetting. Furthermore, to analyze the chemical interactions governing PA adsorption under different states, PA retention was tests for 120 h (Figure S6). DOPBI, which possesses a high BET specific surface area and pore volume, exhibited lower PA retention (59.89%) than that of the others (POPBI and NOPBI, 76.50% and 59.89%). The strong and dense hydrogen bonds exist between the molecular chains of NOPBI. In the stage of PA doping, a significant amount of energy must be expended to break these ordered interchain hydrogen bond networks; this renders the overall reaction thermodynamically unfavorable, thereby limiting absorption. In contrast, in POPBI and DOPBI, electrostatic repulsion prevents the formation of intermolecular hydrogen bonds between polymers, leaving the N and N‐H sites on the imidazole rings in a ‘suspended’ or ‘exposed’ state that is easily accessible. PA molecules do not need to overcome a significant dissociation energy barrier, directly validating the chemical coordination interaction mechanism. The decisive factor in PA adsorption is the chemical coordination interaction at active sites, rather than merely the physical specific surface area. Overall, these results demonstrate that regulating the protonation state of OPBI provides an effective route to optimize both PA uptake and proton transport in the binder phase.

### 2.3 Macroscopic Characterization

To elucidate how the protonation state of OPBI affects catalyst‐ink colloidal behavior, we combined dynamic light scattering (DLS), zeta‐potential analysis, SEM observation, and sedimentation tests [38]. As shown in Figure 3a, the POPBI‐containing ink exhibits a relatively narrow, essentially unimodal particle‐size distribution centered at 306.8 nm (PDI = 26%), whereas the NOPBI ink displays a bimodal distribution with populations at 77.4 and 424.3 nm (PDI = 28%). In contrast, the DOPBI ink is dominated by much larger aggregates with an average size of 1216 nm despite a seemingly lower PDI (19%), consistent with its rapid phase separation. For comparison, bare Pt/C shows a characteristic size of 188.7 nm (PDI = 20%) (Figure 3c), while the corresponding polymer‐only dispersions have average sizes of 160.5, 71.4, and 541 nm for POPBI, NOPBI, and DOPBI, respectively (Figure 3b). Upon mixing with Pt/C, all binders show an increase in hydrodynamic size, indicating the formation of catalyst–binder composite entities rather than simple coexistence of isolated polymer chains and catalyst particles. The relatively narrow POPBI distribution indicates a more homogeneous catalyst–binder association. In contrast, the smaller NOPBI population at 77.4 nm is close to the size of the polymer‐only NOPBI dispersion (71.4 nm), suggesting that a fraction of NOPBI remains free or only weakly associated with Pt/C, while the larger population corresponds to catalyst–binder aggregates.

**FIGURE 3:**
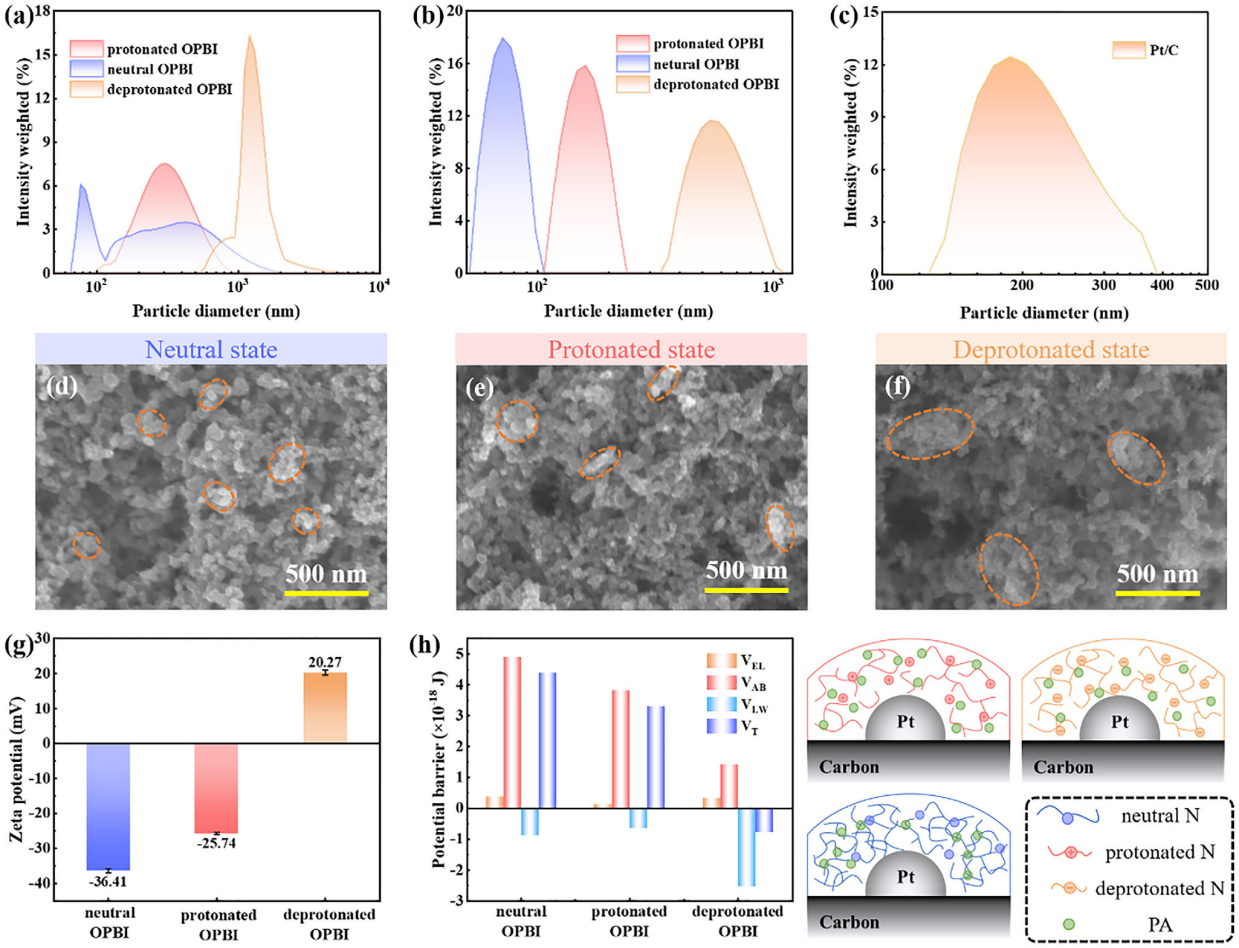
Physical characterization of catalyst inks based on OPBI with different states. (a) Particle size distribution of catalyst ink measured by dynamic light scattering (DLS); (b) Size distribution of OPBI polymer without catalyst particles dispersed in isopropanol/water solution; (c) Particle size distribution of catalyst; (d–f) Scanning electron microscopy (SEM) images of CLs using OPBI binders in neutral, protonated, and deprotonated states, respectively. (g) Zeta potential values of catalyst inks containing OPBI in three states; (h) V_T_ (total potential barrier) and various potential barriers of different OPBI in three states and schematic illustration of OPBI chain configurations in CL under different states.

SEM and TEM observations further support these differences in catalyst–binder organization (Figure 3d–f and Figure S7). POPBI is distributed relatively uniformly within the catalyst layer, whereas NOPBI exhibits localized binder‐rich domains, consistent with incomplete incorporation of part of the binder into the Pt/C network inferred from DLS. DOPBI shows a more pronounced morphological disruption, with large aggregates and an excessively thick binder coating around catalyst particles. Thus, the principal difference between POPBI and NOPBI lies in the uniformity of catalyst–binder association rather than severe macroscopic aggregation, whereas DOPBI undergoes substantial colloidal destabilization. The large DOPBI aggregates and rapid sedimentation are consistent with deprotonation‐induced changes in chain solvation and interfacial interactions. Because DOPBI behaves as an anionic polyelectrolyte, nonuniform polymer adsorption may additionally promote interparticle bridging and flocculation, although this mechanism cannot be established from DLS alone. Zeta‐potential measurements further support these trends while indicating that ink stability is not governed by electrostatic repulsion alone (Figure 3g). NOPBI shows the most negative zeta potential (−36.41 mV), followed by POPBI (−25.76 mV), whereas DOPBI displays an apparent positive value (+20.27 mV), likely related to the presence of counterion‐associated deprotonated chains at the aggregate surface. The macroscopic stability of both POPBI and NOPBI despite their different zeta potentials indicates that surface charge alone cannot account for the observed colloidal behavior; chain conformation, solvation, and catalyst–binder interactions also contribute [39]. This interpretation is corroborated by sedimentation tests (Figure S8), in which POPBI and NOPBI remain macroscopically stable over time, whereas DOPBI undergoes rapid stratification.

To further quantify the origin of these stability differences, the interaction‐energy components within the X‐DLVO framework were calculated at a characteristic separation distance of h = 1 nm (Figure 3h), including Lewis acid–base (V_AB_), electrostatic double‐layer (V_EL_), and van der Waals (V_LW_) interactions [40]. Although the VEL of DOPBI is comparable to that of NOPBI (3.40 × 10^−19^ and 3.84 × 10^−19^ J, respectively), its total interaction energy (V_T_) becomes negative (−7.80 × 10^−19^ J). The V_AB_ of DOPBI (1.41 × 10^−18^ J) is only 29% of that of NOPBI (4.89 × 10^−18^ J), while its V_LW_ attraction increases markedly to −2.53 × 10^−18^ J, together resulting in the collapse of V_T_. By contrast, NOPBI and POPBI exhibit strong V_AB_ repulsion and positive V_T_ values of +4.39 × 10^−18^ and +3.31 × 10^−18^ J, respectively, consistent with their greater colloidal stability. These results directly demonstrate protonation‐state‐dependent differences in colloidal organization, with the most pronounced destabilization occurring for DOPBI.

The resulting catalyst‐layer morphologies provide structural context for the electrochemical behavior discussed below. A more homogeneous catalyst–binder association, as observed for POPBI, is expected to favor continuous binder coverage without excessive local accumulation [41]. In NOPBI, free or weakly associated binder species and localized binder‐rich domains may disrupt proton‐conducting continuity and reduce uniform catalyst utilization without necessarily blocking the overall gas‐transport network. In contrast, the severe aggregation and thick binder coverage observed for DOPBI are expected to impair both pore accessibility and catalyst exposure. These structure–function relationships are proposed from the combined morphological and electrochemical evidence rather than inferred from the ink characterization alone.

### 2.4 Fuel Cell Performance

To establish how protonation‐state regulation of OPBI binders translates into electrode‐level functionality, the electrochemical properties of MEAs prepared with POPBI, NOPBI, DOPBI, and PTFE binders were systematically compared [24, 42, 43]. As shown in Figure 4a–d, the POPBI‐based MEA exhibits the most favorable overall balance among proton transport, oxygen accessibility, and catalyst utilization. Specifically, POPBI delivers the lowest CL proton transport resistance (R_H_^+^, 1.02 Ω cm^2^), together with relatively low ohmic (3.28 Ω cm^2^) and charge‐transfer (1.432 Ω cm^2^) resistances. Its ECSA reaches 217.3 cm^2^ mg_Pt_^−1^, approximately twice that of NOPBI (106.9 cm^2^ mg_Pt_^−1^), while the Pt utilization increases to 25.0% compared with 12.28% for NOPBI (Figure 4e). Importantly, despite their distinct catalyst–binder association, POPBI and NOPBI exhibit similar OTR values of 1519 and 1593 s m^−1^, respectively, differing by less than 5%. This indicates that the localized binder heterogeneity in NOPBI does not substantially impair overall oxygen transport and that enhanced gas diffusion is not the primary origin of the superior ECSA and Pt utilization of POPBI. Instead, NOPBI exhibits a substantially higher R_H_^+^ (1.68 Ω cm^2^), ohmic resistance (4.81 Ω cm^2^), and charge‐transfer resistance (3.4185 Ω cm^2^). Together with the enhanced PA uptake of POPBI established above, these results indicate that the major distinction between POPBI and NOPBI lies in protonic connectivity and catalyst accessibility rather than gas transport. The free or weakly associated binder species and localized binder‐rich domains observed for NOPBI may interrupt effective proton‐conducting pathways and produce nonuniform catalyst utilization, consistent with its higher R_H_^+^, lower ECSA, and lower Pt utilization. This morphology–electrochemistry relationship is considered a mechanistic interpretation supported by the combined DLS, TEM, and electrochemical results rather than a direct consequence established by morphology alone.

**FIGURE 4:**
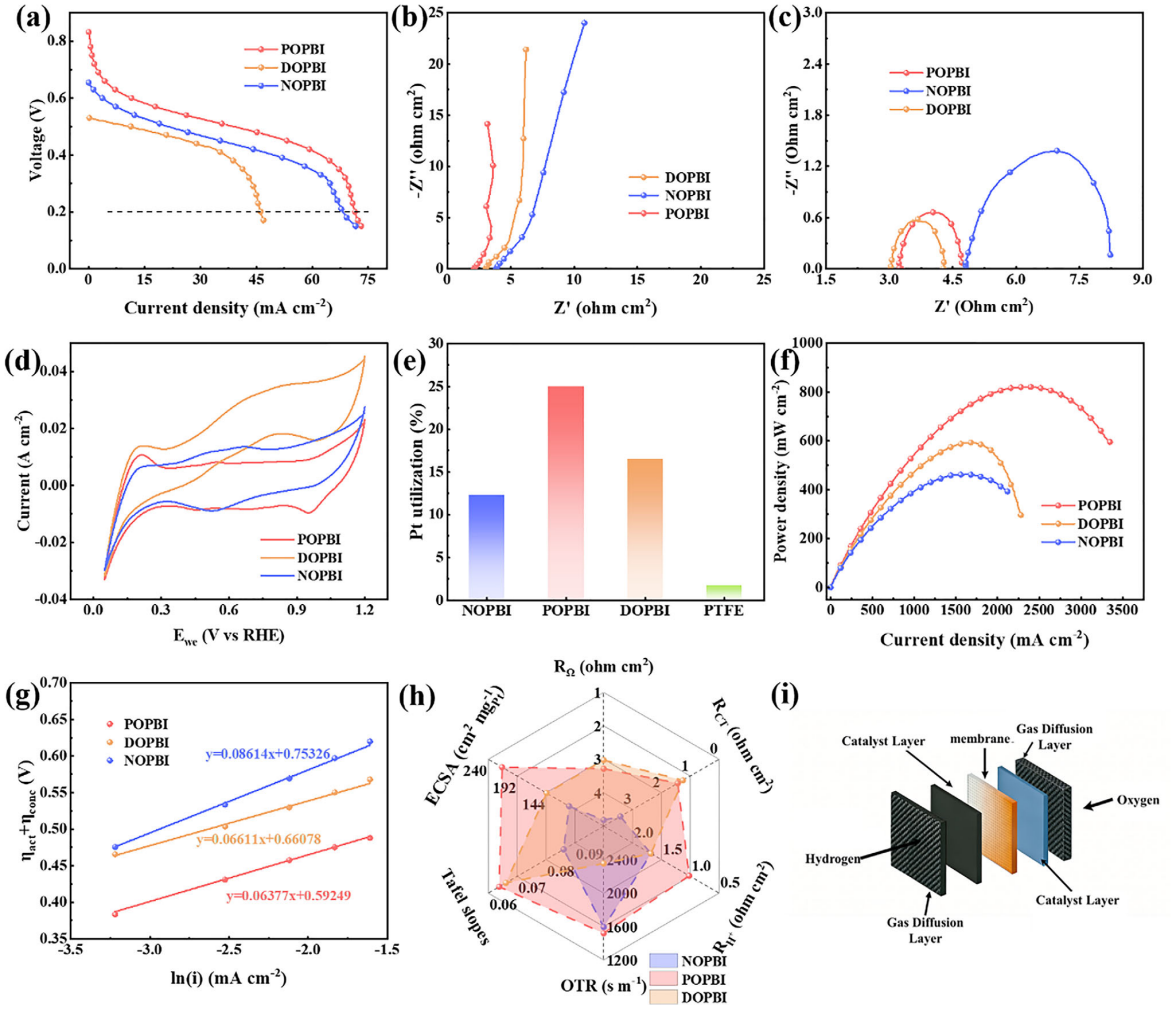
Electrochemical and fuel cell performance of CLs based on OPBI with different states at 160°C. (a) oxygen transport resistance (OTR); (b) Proton conduction resistance (R_H_^+^); (c) Electrochemical impedance spectroscopy (EIS); (d) ECSA; (e) Pt utilization; (f) Power density curves of CLs with X‐OPBI; (g) Fitting results of Tafel slopes; (h) Comparison chart of various performances; and (i) Schematic diagram of MEA structure.

DOPBI exhibits a distinctly different limitation. Although the DOPBI‐based MEA shows the lowest ohmic resistance (3.02 Ω cm^2^) and charge‐transfer resistance (1.255 Ω cm^2^), its OTR increases sharply to 2353 s m^−1^, while its ECSA and Pt utilization remain limited to 143.9 cm^2^ mg_Pt_^−1^ and 16.54%, respectively. Unlike the localized binder heterogeneity of NOPBI, the large‐scale aggregation and excessive binder coverage of DOPBI are therefore accompanied by a substantial oxygen‐transport penalty. Together with the morphology observations, these results are consistent with restricted pore accessibility and excessive catalyst coverage in the DOPBI‐based CL. Such a structure may partially lower local ionic or interfacial resistance, yet simultaneously impair oxygen diffusion and active‐site exposure. Thus, the electrochemical consequences of binder heterogeneity depend strongly on its mode and length scale: localized nonuniform association in NOPBI primarily affects protonic continuity and catalyst utilization, whereas severe aggregation and excessive coverage in DOPBI additionally impose pronounced oxygen‐transport limitations.

The PTFE‐based electrode further underscores the importance of a proton‐conducting binder in this system. Despite providing structural integrity, PTFE exhibits the highest ohmic resistance (9.48 Ω cm^2^), the largest charge‐transfer resistance (5.00 Ω cm^2^), and extremely low ECSA and Pt utilization (1.70%), confirming the intrinsic limitation of a non‐proton‐conducting binder in constructing an efficient reaction environment in PA‐PBI‐based HT‐PEMFC CLs. Consequently, the favorable balance among proton transport, catalyst accessibility, and oxygen transport enables the POPBI‐based MEA to deliver the highest peak power density of 820 mW cm^−2^ at 160°C under anhydrous conditions, 1.78 times that of the NOPBI‐based MEA (Figure 4f and Figure S9). Notably, the complete electrochemical dataset indicates that the superiority of POPBI does not arise from minimizing every individual resistance component, but from achieving a balanced reaction environment within the CL.

The kinetic characteristics of the different electrodes provide further support for this structure–function relationship. As shown in Figure 4g, the POPBI‐based MEA shows the lowest Tafel slope (63.77 mV dec^−1^), indicating reduced apparent kinetic polarization and a more favorable local reaction environment for the oxygen reduction reaction. Because the Pt catalyst itself is unchanged, this improvement is more reasonably attributed to enhanced proton accessibility and catalyst utilization than to a fundamental change in the intrinsic activity of Pt. XPS analysis (Figure S10) shows a modest 0.07 eV negative shift in the Pt 4f binding energy for Pt‐POPBI, suggesting a slightly altered interfacial electronic environment [44]. Although this shift is small, it is consistent with binder‐mediated interfacial interactions that may facilitate charge transfer. The overall performance comparison is summarized in Figure 4h, and a schematic illustration of the MEA structure is shown in Figure 4i.

Taken together, these results establish a multiscale structure–property–performance relationship. Experimentally, protonation‐state regulation alters PA uptake, catalyst–binder organization, and binder distribution, accompanied by distinct proton transport, ECSA, Pt utilization, and, in the case of severe DOPBI aggregation, oxygen transport. Based on the combined evidence, we propose that POPBI benefits from enhanced PA‐assisted proton transport and more favorable binder organization, which jointly improve catalyst accessibility without substantially compromising oxygen transport. Thus, protonation‐state engineering simultaneously regulates proton transport and catalyst–binder organization within the CL. The similar protonation‐state‐dependent trend observed for mPBI (Figure S11) further suggests that this strategy may represent a general design principle for PBI‐based binders.

To further optimize the proton‐conducting environment within the CL, PA was directly introduced into the POPBI ink, and the resulting electrodes were evaluated electrochemically [12]. As shown in Figure 5a and Figure S12, the CL containing an intermediate PA content (POPBI‐20PA) exhibits the best overall performance, delivering the highest peak power density of 912 mW cm^−2^, the lowest ohmic and charge‐transfer resistances (2.15 and 0.98 Ω cm^2^, respectively), and the highest ECSA (Figure S13a,b). These results clearly indicate the existence of an optimal acid‐loading window in the CL. At insufficient PA content, proton percolation within the electrode is incomplete, limiting proton accessibility to catalytic sites. By contrast, moderate PA addition improves protonic continuity and facilitates triple‐phase‐boundary formation, thereby enhancing catalyst utilization and overall reaction efficiency. However, excessive PA leads to an increase in OTR (Figure S13c), indicating that overabundant acid phases impede oxygen transport and may partially block catalytic sites [45]. Thus, PA acts not only as a proton‐conducting medium but also as a transport‐modulating phase, and optimal performance is achieved only when proton transport enhancement is balanced against gas‐transport penalties. This balance becomes even more important under Pt‐lean conditions, where device performance increasingly depends on how efficiently each Pt site participates in the reaction [46, 47, 48]. As shown in Figure 5b,c and Figures S14 and S15, the Pt mass‐specific output varies strongly with Pt loading. At a Pt loading of 0.11 mg cm^−2^, the electrode achieves a Pt mass‐specific peak power density of 3.38 W mg_Pt_^−1^. More importantly, when the Pt loading is decreased to an ultralow value of 0.075 mg cm^−2^, the POPBI‐20PA‐based MEA delivers an outstanding Pt mass‐specific peak power density of 5.25 W mg_Pt_^−1^ at a gas backpressure of 1.5 bar. This value exceeds the range reported for previously reported PA‐PBI‐based HT‐PEMFCs (1.08–4.14 W mg_Pt_^−1^), as benchmarked in Figure 5d and Table S1, placing the present system among the best‐performing PBI‐binder‐based HT‐PEMFC electrodes reported to date [20, 21, 22, 23, 24, 25, 49, 50, 51].

**FIGURE 5:**
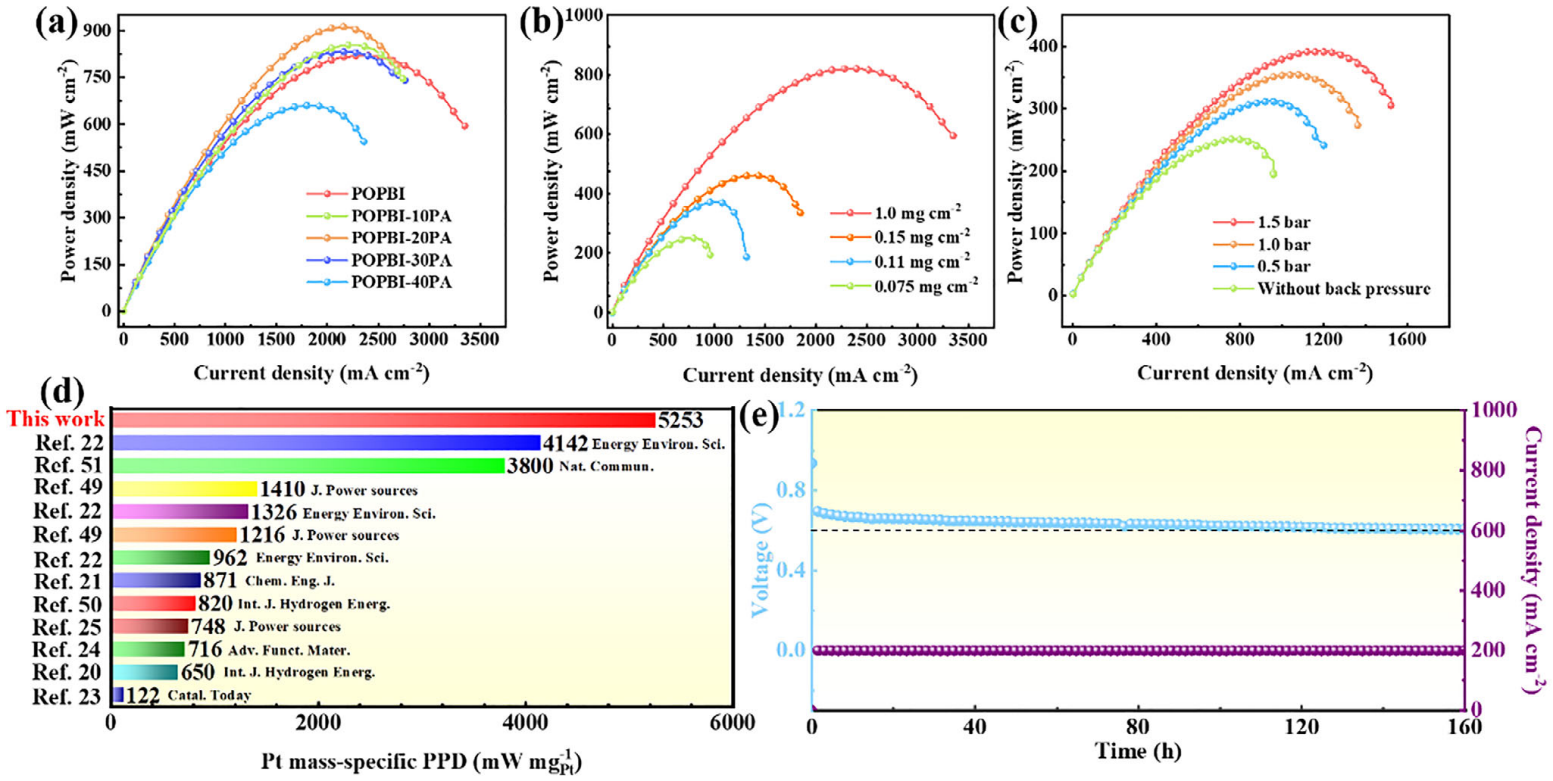
Fuel cell performance of CLs with POPBI at 160°C. (a) Performance of POPBI‐based CLs with varying PA content; (b) Effect of Pt loading on fuel cell performance; (c) Performance under different backpressures for POPBI‐20PA‐CL with a Pt loading of 0.075 mg cm^−2^; (d) Comparison of peak power density for representative PA‐doped PBI‐based HT‐PEMFCs at 160°C [20, 21, 22, 23, 24, 25, 49, 50, 51]; (e) Long‐term durability test of POPBI‐20PA‐CL at 200 mA cm^−2^.

The strong mass‐specific output under ultralow Pt loading further highlights the central role of protonation‐state engineering. When Pt inventory is reduced, the decisive factor is no longer the total amount of catalyst, but the fraction of Pt sites that are simultaneously accessible to protons, reactant gases, and electrons. The superior performance of POPBI under these conditions therefore confirms that protonated OPBI maximizes triple‐phase‐boundary efficiency and minimizes inactive Pt, allowing the CL to maintain high reaction efficiency even at very low noble‐metal loading. Finally, the long‐term stability test (Figure 5e) shows that the fuel‐cell voltage remains above 600 mV over extended operation. Furthermore, the CL was characterized following long‐term testing, and it was found that there was essentially no change in the catalyst morphology, particle size or size distribution (Figure S16). In the FT‐IR spectrum, the peak at 1637 cm^−1^ corresponds to C═O, indicating that the OPBI polymer underwent slight oxidation during the long‐term test, but the damage was not significant and the main structure remained intact. It demonstrates that the protonated OPBI binder not only enhances initial performance but also maintains a sufficiently robust CL structure for durable HT‐PEMFC operation.

## 3 Conclusion

In summary, this work demonstrates protonation‐state engineering as a simple and structure‐preserving strategy for optimizing commercial OPBI binders in HT‐PEMFC catalyst layers. By comparing protonated, neutral, and deprotonated OPBI binders, we show that formic‐acid protonation generates benzimidazolium sites that loosen chain packing, enlarge interchain spacing, and enhance PA uptake without altering the polymer backbone. Consequently, the protonated OPBI binder achieves a PA uptake increased by 382% and a proton conductivity of 357 mS cm^−1^, while forming a homogeneous catalyst–binder assembly that enriches triple‐phase boundaries, reduces CL proton‐transport resistance by 63.7%, and doubles ECSA and Pt utilization relative to neutral OPBI. The optimized MEA delivers a peak power density of 912 mW cm^−2^ and a Pt‐mass‐specific peak power density of 5.25 W mg_Pt_^−1^ at an ultralow cathode Pt loading of 0.075 mg cm^−2^. These results establish protonation‐state control as an accessible route to high‐performance PBI‐based binders for Pt‐lean HT‐PEMFCs.

## 4 Experiment

### 4.1 Materials

3,3'‐diaminobenzidine (97%) was purchased from TCI Development Co.; Methane sulfonic acid (MSA, 98%) was purchased from Aladdin Holdings Group Co., Ltd; Ethanol absolute (AR) was purchased from Shenzhen Mingke Technology Co., Ltd; PA (85%) was purchased from Shanghai Macklin Biochemical Co., Ltd; Sodium bicarbonate (NaHCO_3_, 99%), dimethyl sulfoxide‐D*_6_* (DMSO‐d*_6_*, 99.8%), 4,4'‐diphenyl ether dicarboxylic acid (98%), isophthalic acid, phosphorus pentoxide (P_2_O_5_, 99%), *N*‐methyl‐pyrrolidone (NMP, 99.5%), isopropanol (IPA), NaOH were purchased from Shanghai Energy Chemical Co., Ltd.; formic acid (FA) was purchased from Tianjin Yongda chemical reagent Co., Ltd; Pt/C catalyst, gas diffusion layer (GDL) were purchased from Suzhou Sinero Technology Co., Ltd.

### 4.2 Synthesis of OPBI and mPBI

The OPBI and mPBI were synthesized according to the literature [52].

### 4.3 Preparation of CL and MEA

OPBI powder was dissolved in various solvents (FA, NMP and 5% NaOH/Ethanol mixture) to prepare a 2 wt.% OPBI solution at 80°C. Catalyst ink was prepared by first adding a minimal amount of deionized water to an appropriate quantity of Pt/C catalyst for infiltration, followed by the addition of IPA as a dispersant and the OPBI solution. After 30 min of ultrasonic treatment, CLs were prepared by spraying this ink onto a GDL substrate at 80°C and then dried under vacuum at 100°C for 2 h. In particular, DOPBI must be soaked for 30 min in 1 M hydrochloric acid and in deionized water respectively. The CLs had Pt loadings of 1.0, 0.15, 0.11, and 0.075 mg cm^−2^, with the OPBI binder content fixed at 2.5 wt.% relative to the Pt/C catalyst. These CLs are designated as XOPBI where X denotes the solvent used (FA (protonated), NMP (neutral), or 5% NaOH/Ethanol mixture (deprotonated)). PA, acting as both a proton conductor and a critical agent for forming TPBs on the catalyst surface [12], was incorporated into the catalyst ink. To investigate the effect of PA content, different quantities of a PA/IPA aqueous solution (PA: IPA aqueous solution = 1:100 wt./wt.; water: IPA = 2:11 wt./wt.) were added to the prepared ink. The resulting CLs are named XOPBI‐YPA, where Y represents the PA mass added (10, 20, 30, or 40 mg). In addition, to explore the influence of different gas backpressure (0.5, 1.0, and 1.5 bar) on CL, POPBI‐20PA with a Pt loading of 0.075 mg cm^−2^ was tested. MEAs were fabricated by hot‐pressing two identical CLs onto either side of an OPBI membrane (thickness about 30 ± 5 µm). Before preparing MEA, all OPBI membranes were doped by soaking in PA at 160°C for 24 h, achieving a doping level of 266 ± 3%. CLs with mPBI binder were prepared using the same procedure.

### 4.4 Characterization

The characterization methods and results are presented in the Supporting Information.

## Author Contributions

**Jinzhen Huang**: conceptualization, validation, data curation, investigation, writing – original draft, software, methodology. **Minhui Liang**: software, data curation. **Jingjing Lin**: funding acquisition, supervision. **Zhihuan Pan**: methodology. **Jinwu Peng**: supervision, funding acquisition, writing – review and editing. **Lei Wang**: writing – review and editing, funding acquisition, supervision.

## Conflicts of Interest

The authors declare no conflicts of interest.

## Supporting information




**Supporting File**: advs78090‐sup‐0001‐SuppMat.docx.

## Data Availability

The data that support the findings of this study are available from the corresponding author upon reasonable request.
