# Supplementary material for: Protonation Modification of Commercial OPBI as Binders Toward High‐Performance High‐Temperature PEM Fuel Cells
Source: Adv Sci (Weinh). 2026 Sep 28:e78090. Online ahead of print. doi: 10.1002/advs.78090 (PMC13620256; doi:10.1002/advs.78090)
Supplement: Supplementary file 1 [file ADVS-9999-e78090-s001.docx]

**Supporting Information**

**Protonation Modification of Commercial OPBI as Binders toward High-Performance High-Temperature PEM Fuel Cells**

Jinzhen Huang^1,2^, , Minhui Liang^1^, Jingjing Lin^1^,Zhihuan Pan^1^, Jinwu Peng^1, *^, Lei Wang^1,2,3, *^

^1^*Guangdong Provincial Key Laboratory of New Energy Materials Service Safety, Shenzhen Key Laboratory of Polymer Science and Technology, College of Materials Science and Engineering, Shenzhen University, Shenzhen, 518060, China*.

^2^ Zhangjiang Laboratory, 100 Haike Road, Pudong, Shanghai 201210, China.

^3^ Key laboratory of Resources Environmental and Green Low Carbon Processes in East Guangdong, Chaozhou, 521041, China.

Corresponding author: Lei Wang, E-mail: [wl@szu.edu.cn](mailto:wl@szu.edu.cn)

*Jinwu Peng, E-mail:* *[pengjw@szu.edu.cn](mailto:pengjw@szu.edu.cn)*

Manuscript submitted to **Advanced Science**

1. **Characterization methods**

The chemical structure of OPBI, mPBI and POPBI were confirmed by ^1^H NMR spectroscopy recorded on Avance Ⅲ 500 (Bruker, Germany) with a frequency of 500 MHz using dimethyl sulfoxide-D*_6_* (DMSO-d*_6_*) as the solvent. And the chemical structure was characterized by a Fourier transform infrared spectrometer (Nicolet 6700, USA), the polymer powder and KBr were mixed at a mass ratio of 1 to 100.

Morphology of the inks and catalyst layers were evaluated by using a high-resolution thermal field emission scanning electron microscope (SU-70).

By measuring the changes of the mass of the OPBI with time, the Acid doping level of the OPBI were obtained. Typically, samples were soaked in 85 wt.% PA at 160 ℃ for 24 h, then took it out, and wiped off the excess PA. the samples were dried in an 80 ℃ oven for 5 h, and the sample weight and size was recorded.

$$Acid uptake (\%)=\frac{w_{2}-w_{1}}{w_{1}}\times100\%$$

where *w*_1_ represent the mass of the dry OPBI, *w*_2_ represent the mass of the PA doped OPBI.

Proton conductivity was measured by an electrochemical workstation (Zahner IM6ex, Germany) using AC impedance mode with a frequency range of 1-10^4^ Hz. The PA doped OPBI was placed into an airtight mold with pure Au electrode. At beginning, the sample was heated at 120 °C for 1.5 h to achieve anhydrous conduction. Then, test the proton conductivity. The temperature range was from 120 °C to 180 °C, and it was balanced for 15 min every 10 °C increase. The proton conductivity (*σ*) was calculated according to follow equation:

$$\sigma=\frac{L}{R\cdot S}$$

where *L*, *R* and *S* represent the distance between the pure Au electrodes, the resistance and cross-sectional area of the samples, respectively.

In situ EIS of membrane electrode assembly was measured using Chenhua CHI660 electrochemical workstation at a frequency of 0.1 to 10^4^ Hz in a constant potential mode of 0.7 V. Other test conditions were the same as those of the fuel cell performance test.

In situ CV was measured at 160 °C with an H_2_ flow of 200 Sccm and an N_2_ flow of 400 Sccm to the anode and cathode, respectively. The CVs were obtained between 0.05 V and 1.20 V. The electrochemical surface area (ECSA) was calculated based on the hydrogen desorption peak.

$$ECSA (cm^{2} {mg}_{Pt}^{-1})=\frac{Q}{0.21\times S \times m}$$

Where Q is the integral of the hydrogen desorption peak in the CV curve, S is scanning rate (0.05 V s^-1^), and m is Pt loading in GDE. The coefficient of hydrogen absorbed by Pt is 0.21 mC cm^-2^.

Pt utilization (%) was calculated from:

$$Pt utilization (\%)=\frac{ECSA (cm^{2} {mg}_{Pt}^{-1})}{Theoretical ECSA value (870 cm^{2} {mg}_{Pt}^{-1})}$$

Pt-mass specific PPD (peak power density) (W mg_Pt_^-1^) was calculated from:

$$\mathrm{Pt}­mass specific PPD \left( W {mg}_{Pt}^{-1} \right)=\frac{Peak Pow density (W{cm}^{­2})}{Pt loading of the cathode ({mg}_{Pt} {cm}^{­2})}$$

In situ proton conductance resistance was measured using Chenhua CHI660 electrochemical workstation at a frequency of 0.1 to 10^4^ Hz in a constant potential mode of 0.7 V. Other test conditions were the same as those of the CV test. Ideally, in the H_2_/N_2_ Nyquist plot, the 45° linear region below the high frequency corresponds to the migration of H^+^ through the ionomer phase in the CL. The lower frequency line, which appears below this region, exhibits a 90° phase angle attributed to the total capacitance. For simplicity, the resistance to proton transport through the CL (𝑅_H_^+^) can be determined from the length of the Warburg-like region (𝑅_H_^+^/3) projected onto the real axis (*Z*′). In practical tests, the curves deviate from the ideal 45° and 90° lines, which can be attributed to the inhomogeneous distribution of the H^+^ resistance within the CL. 𝑅_H_^+^/3 is obtained by extrapolating an asymptote from the low-frequency line on *Z*′ and measuring the intersection point, from which 𝑅_H_^+^ can be derived.

The porous properties of OPBIs were clarified by nitrogen adsorption and desorption isotherms measured at 77 K (Quanta chrome). Samples were de-gassed at 200 °C for 12 h under vacuum before analysis. The surface areas were calculated in the relative pressure (P/P_0_) range from 0.01 to 0.1.

Fuel cell performance was appraised by single-cell test at 160 °C under anhydrous condition. The membrane-electrode assemblies were prepared by pressing anode and cathode onto both sides of proton exchange membranes. Setting gas feeding rates for hydrogen and oxygen were 80 and 160 Sccm, using a multiple independent channel test station (Arbin® MITS PRO 7.0) to record the experiment data.

The method of measuring total oxygen resistance (R_tot_) based on the study on a previous study. In fuel cell operation, the limiting current density (I_lim_) represents the maximum achievable current density when oxygen transport resistance becomes the rate-limiting factor for electrochemical reactions. To systematically characterize oxygen transport limitations, oxygen dilution experiments are typically conducted using nitrogen diluted oxygen mixtures. Under this condition, the oxygen concentration at catalytic reaction sites approaches depletion (near-zero concentration), rendering further cathode potential reduction ineffective for current enhancement. The limiting-current measurement was performed using H_2_ (80 Sccm) and diluted O_2_ (160 Sccm) without humidification. To confirm that limiting current density (I_lim_) is completely controlled by the oxygen mass transport polarization, 1% O_2_ with balance gas of nitrogen is used in this experiment. A polarization (V–I) curve was measured at 160 °C by sweeping the potential from open circuit to 0.15 V, and record a point every 10-mV drop. In the resulting curve, the values of the current at 0.2 V were employed as I_lim_. The R_tot_ is represented as follows:

$$R_{tot}(s m^{-1}) =\frac{4FPX_{O_{2}}}{RTI_{lim}}$$

Where *F* is the Faraday constant (96485 C mol^−1^), *P* is the pressure, $X_{O_{2}}$ is the molar fraction of oxygen, R is the ideal gas constant (8.314 J mol^-1^ K^-1^), T (K) is the test temperature. Convert the final unit to s m^-1^.

SmartLab (Rigaku company, Japan) was used for X-ray diffraction (XRD) testing. The testing voltage and current are 40 kV and 200 mA, respectively. The X-ray used is Cu kα (λ) = 1.5418 Å). The range of 2*θ* is 10-80° (step size is 0.02°).

$$d=\frac{\lambda}{2sin\theta}$$

Where, *d* is chain spacing (d-spacing), $\lambda$ is X-ray wavelength (Average wavelength of copper target Kα, *λ*=1.5418 Å), $\theta$ is the angle of incidence ($2\theta$ is the diffraction angle measured by the diffractometer).

The particle size and Zeta potential of the catalyst ink were tested using a nanoparticle size detector (ZetaPlus, Austria).

The surface valence state of the membrane was tested by an X-ray photoelectron spectrometer (Thermo Scientific K-Alpha, USA).

The contact Angle between the membrane and PA was tested by a dynamic contact Angle tester (Dataphysics /OCA 25, Germany).

The thermal stability of the polymer was tested by the thermogravimetric analyzer (TGA Q50, USA). The specific test procedure is as follows: Raise the temperature from room temperature to 120 °C at a rate of 10 °C min^-1^ and maintain it for 10 min. Then reduce it to 100 °C at a rate of 10 °C min^-1^ and maintain it for 10 min. Finally, increase the temperature to 800 ℃ at a rate of 10 °C min^-1^.

The Chain conformation and aggregation state of the OPBI were tested by ultraviolet/visible/near-infrared spectrophotometer (PerkinElmer Lambda 950, China).

**Computational methods**

The density functional theory (DFT) calculations were performed using the DMol3.^[1]^ All structures were fully optimized with the following convergence criteria: energy tolerance of 1×10^-5^ Ha, maximum force threshold of 0.002 Ha/Å, and atomic displacement tolerance of 5×10^-3^ Å. The exchange-correlation interactions were modeled using the hybrid B3LYP functional,^[2, 3]^ and the van der Waals dispersion corrections were incorporated via the Tkatchenko-Scheffler (TS) method to account for non-covalent interactions.^[4]^ Spin polarization was explicitly included in all calculations, and a global charge compensation scheme was applied to ensure neutrality for charged systems.^[5]^ The electronic wavefunctions were expanded with the double numerical plus d-polarization (DND) basis set (basis file version 3.5), and all-electron treatment was adopted to rigorously describe core-electron interactions.

All DFT calculations were performed with Gaussian 16, Rev. A03 programs.^[6]^ The B3LYP hybrid functional^[7, 8]^ including Grimme empirical dispersion correction (D3BJ)^[9, 10]^ with 6-31G(d) basis set^[11]^ were used for the geometry optimization of all structures. Harmonic vibrational frequency analysis was calculated at the same level to verify that geometric structure has positive frequency. The B3LYP hybrid functional with def2-tzvp^[12, 13]^ basis set were used to calculated energy of all structures.

The calculations and visualization of IGMH were performed by Multiwfn^[14]^ and VMD^[15]^ software.

To elucidate the colloidal stability mechanism of catalyst inks containing different polymeric binders, we employed the Extended Derjaguin–Landau–Verwey–Overbeek (X-DLVO) theory. The total interaction potential energy (V_T_) between two spherical particles as a function of separation distance (h) is expressed as the sum of Lifshitz-van der Waals (V_LW_), electrostatic double-layer (V_EL_), and Lewis acid-base (V_AB_) interactions:

$$V_{T}\left( h \right)=V_{Lw}\left( h \right)+V_{EL}\left( h \right)+V_{AB}(h)$$

Under the Derjaguin approximation for two identical spheres of radius R (where h≪ R), the respective components are given by:

$$V_{Lw}\left( h \right)=-\frac{A\times R}{12h}$$

$$V_{EL}\left( h \right)=2\pi\varepsilon\varepsilon_{0}R\psi_{0}^{2}ln[1+exp(-\kappa h)]$$

$$V_{AB}\left( h \right)=2\pi R\lambda{\Delta G}_{h_{0}}^{AB}exp(\frac{h-h_{0}}{\lambda})]$$

Where A is the Hamaker constant, εε_0_ is the permittivity of the medium, ψ_0_ is the surface potential (approximated by the Zeta potential), κ−1 is the Debye screening length, λ is the decay length of water molecules (0.6 nm), h_0_=0.158 nm is the minimum contact distance, and ΔG_h0_^AB^ is the Lewis acid-base interaction free energy at contact.

Due to the swelling effect of diiodomethane on the polymer binder films, a complete three-liquid contact angle approach was not feasible. Instead, we adopted a semi-quantitative approach based on the van Oss-Chaudhury-Good (vOCG) equation [2] using water as the probe liquid.

For a solid-liquid system, the vOCG equation is:

$$\gamma_{L}(1+\cos\theta)=2(\sqrt{\gamma_{S}^{LW}\gamma_{L}^{LW}}+\sqrt{\gamma_{S}^{+}\gamma_{L}^{-}}+\sqrt{\gamma_{S}^{-}\gamma_{L}^{+}}$$

Where γ^LW^, γ^+^, and γ^−^ represent the apolar Lifshitz-van der Waals component, Lewis acid (electron acceptor), and Lewis base (electron donor) components, respectively.

For the present carbon-based catalyst system:

(i) The apolar component is taken from the literature as γ_S_^LW^=45.0 mJ m^−2^.
(ii) Carbon surfaces lack strong electron-accepting sites; hence, γ_S_^+^ ≈ 0 mJ m^−2^.

For water (probe liquid) at 25 °C, the known parameters are:

γ_L_ = 72.8, γ_L_^LW^ = 21.8, γ_L_^+^ = 25.5, γ_L_^−^ = 25.5, γ_S_^+^ = 0, γ_L_^+^ = 25.5 (all in mJ·m^−2^).

$$\sqrt{\gamma_{s}^{-}}=\frac{\gamma_{L}(1+cos\theta)-2\sqrt{\gamma_{S}^{LW}\gamma_{L}^{LW}}}{2\sqrt{\gamma_{L}^{+}}}$$

Calculation of the Lewis Acid-Base Interaction Free Energy (ΔG_h0_^AB^)

The polar (AB) interaction free energy at the minimum contact distance (h_0_ = 0.158 nm) is calculated using the two surface components (solid S and liquid L) [1]:

$${\Delta G}_{h_{0}}^{AB}=-2(\sqrt{\gamma_{S}^{+}}-\sqrt{\gamma_{L}^{+}})(\sqrt{\gamma_{S}^{-}}-\sqrt{\gamma_{L}^{-}})$$

γ_S_^+^ = 0, $\sqrt{\gamma_{L}^{+}}=5.05$, and $\sqrt{\gamma_{L}^{-}}=5.05$

2. **Characterization results**

**Figure S1** Synthesis of (a) mPBI and (b) OPBI, (c) ^1^H NMR and (d) FT-IR of OPBI and mPBI

**Figure S2** Thermal stability of OPBIs in different states.

**Figure S3** FT-IR of OPBI in different states.

**Figure S4** (a) N_2_ adsorption–desorption isotherms of NOPBI, POPBI and DOPBI at 77.3 K, (b) incremental pore volumes of NOPBI, POPBI and DOPBI as a function of the pore size measured from N_2_ adsorption–desorption isotherms.

**Figure S5** The contact angle between the different binders and PA.

**Figure S6** PA remain of OPBI binders in neutral, protonated, and deprotonated states, respectively.

**Figure S7** TEM images of CLs using OPBI binders in neutral, protonated, and deprotonated states, respectively.

**Figure S8** Visual inspection of the settling behavior of the standard ink by different OPBIs (a) the ink in preparing, (b) preparation for 1 h (From left to right, they are NOPBI, POPBI and DOPBI).

**Figure S9** Polarization curves of CLs with X-OPBI.

**Figure S10** XPS Pt 4f spectra of different CL.

**Figure S11** The fuel cell performance of CL prepared with different mPBIs at 160 °C.

**Figure S12** Performance of POPBI-based CLs with varying PA content

**Figure S13** The fuel cell performance of CL prepared with different PA contents. (a) OTR, (b) EIS, and (c)ECSA of MEA with POPBI-YPA at 160 °C.

**Figure S14** Effect of Pt loading on fuel cell performance.

**Figure S15** Performance under different backpressures for POPBI-20PA-CL with a Pt loading of 0.075 mg cm^-2^.

**Figure S16** SEM and TEM image of POPBI-20PA-CL. (a, c) before the long-term test, (b, d) after the long-term test.

**Figure S17** FT-IR of POPBI and NOPBI before and after the long-term test.

**Table S1** Summary and comparison of the performance of representative PA-PBI-based HT-PEMFC at 160 °C.

| **Binder** | **Solvent** | **Test conditions** | **PPD**  **(mW cm^-2^)** | **Pt loading (mg cm^-2^)** | **Pt mass-specific PPD**  **(mW mg-1 Pt)** | **Ref.** |
| --- | --- | --- | --- | --- | --- | --- |
| PBI | DMAc | 200 mL min^-1^ (H_2_), 1000 mL min^-1^ (air) | 325 | 0.5 | 650 | [16] |
| mPBI | NMP | H_2_ and O_2_ with a flow rate 200 sccm | 608 | 0.5 | 1216 | [17] |
| F6PBI | NMP | H_2_ and O_2_ with a flow rate 200 sccm | 705 | 0.5 | 1410 | [17] |
| F4PBI | NMP | H_2_ and O_2_ with a flow rate 200 sccm | 648 | 0.5 | 1296 | [17] |
| PPO-HPA^a^ | DMSO | 100 mL min^-1^ (H_2_), 300 mL min^-1^ (air) | 122 | 1.0 | 122 | [18] |
| PBI^b^ | DMAc | 134 mL min^-1^ (H_2_), 400 mL min^-1^ (air) | 540 | 0.5 | 1080 | [19] |
| PFPA-PIM-SBI | DMSO | H_2_ and O_2_ with flow rate 0.5 L min^-1^ | 663 | 0.5 | 1326 | [20] |
| PFPA-PIM-SBI | DMSO | H_2_ and O_2_ with flow rate 0.5 L min^-1^ | 290 | 0.07 | 4142 | [20] |
| mPBI | DMSO | H_2_ and O_2_ with flow rate 0.5 L min^-1^ | 481 | 0.5 | 962 | [20] |
| OPBI | NMP | 80 sccm (H_2_), 160 sccm (O_2_) | 461 | 1.0 | 461 | This work |
| OPBI | NaOH/Ethanol | 80 sccm (H_2_), 160 sccm (O_2_) | 592 | 1.0 | 592 | This work |
| OPBI | FA | 80 sccm (H_2_), 160 sccm (O_2_) | 912 | 1.0 | 912 | This work |
| OPBI | FA | 80 sccm (H_2_), 160 sccm (O_2_) | 461 | 0.15 | 3073 | This work |
| OPBI | FA | 80 sccm (H_2_), 160 sccm (O_2_) | 373 | 0.11 | 3391 | This work |
| OPBI^c^ | FA | 80 sccm (H_2_), 160 sccm (O_2_) | 394 | 0.075 | 5253 | This work |

^a^ Test temperature is 150 °C

^b^ Test temperature is 125 °C

^c^ Back pressure is 1.5 bar

**References**

[1] B. Delley, From molecules to solids with the DMol 3 approach, The Journal of chemical physics, 113 (2000) 7756-7764.

[2] A.D. Becke, Density‐functional thermochemistry. III. The role of exact exchange, The Journal of chemical physics, 98 (1993) 5648-5652.

[3] P.J. Stephens, F.J. Devlin, C.F. Chabalowski, M.J. Frisch, Ab initio calculation of vibrational absorption and circular dichroism spectra using density functional force fields, The Journal of physical chemistry, 98 (1994) 11623-11627.

[4] A. Tkatchenko, M. Scheffler, Accurate Molecular Van Der Waals Interactions from Ground-State Electron Density and Free-Atom Reference Data, Physical review letters, 102 (2009) 073005.

[5] G. Kresse, J. Furthmüller, Efficient iterative schemes for ab initio total-energy calculations using a plane-wave basis set, Physical review B, 54 (1996) 11169.

[6] Frisch, M. J.; Trucks, G. W.; Schlegel, H. B., et al. Gaussian 16 Rev. A.03, Wallingford, CT, 2019.

[7] P.J. Stephens*, F.J. Devlin, C.F. Chabalowski, M.J. Frisch, Ab Initio Calculation of Vibrational Absorption and Circular Dichroism SpectraUsing Density Functional Force Fields, J Phys Chem, 98 (1994) 11625.

[8] A.D. Becke, Density-functional thermochemistry. III. The role of exact exchange, J Chem Phys, 98 (1993) 5648-5652.

[9] S. Grimme, J. Antony, S. Ehrlich, H. Krieg, A consistent and accurateab initioparametrization of density functional dispersion correction (DFT-D) for the 94 elements H-Pu, J Chem Phys, 132 (2010) 154104.

[10] S. Grimme, S. Ehrlich, L. Goerigk, Effect of the damping function in dispersion corrected density functional theory, J Comput Chem, 32 (2011) 1456-1465.

[11] R. Krishnan, J.S. Binkley, R. Seeger, J.A. Pople, Self-consistent molecular orbital methods. XX. A basis set for correlated wave functions, J Chem Phys, 72 (1980) 650-654.

[12] F. Weigend, R. Ahlrichs, Balanced basis sets of split valence, triple zeta valence and quadruple zeta valence quality for H to Rn: Design and assessment of accuracy, Phys Chem Chem Phys, 7 (2005) 3297-3305.

[13] J. Tomasi, B. Mennucci, R. Cammi, Quantum mechanical continuum solvation models, Chem Rev, 105 (2005) 2999-3093.

[14] W. Humphrey, A. Dalke, K. Schulten, VMD: Visual molecular dynamics, J Mol Graph, 14 (1996) 33-38.

[15] T. Lu, F.W. Chen, Multiwfn: A multifunctional wavefunction analyzer, Journal of Computational Chemistry, 33 (2012) 580-592.

[16] H. Su, S. Pasupathi, B. Bladergroen, V. Linkov, B.G. Pollet, Optimization of gas diffusion electrode for polybenzimidazole-based high temperature proton exchange membrane fuel cell: Evaluation of polymer binders in catalyst layer, Int J Hydrogen Energ, 38 (2013) 11370-11378.

[17] G. Chao, H. Tang, Q. Ju, N. Li, K. Geng, Fluorinated polybenzimidazole as binders for high-temperature proton exchange membrane fuel cells, J Power Sources, 556 (2023) 232473.

[18] D.-H. Kim, C.-M. Min, E. Leea, J.-S. Lee, C. Pak, Effect of vinylphosphonic acid and polymer binders with phosphate groups on performance of high-temperature polymer electrolyte membrane fuel cell, Catal Today, 358 (2020) 333–337.

[19] J. Lobato, P. Can˜izares, M.A. Rodrigo, J.J. Linares, F.J. Pinar, Study of the influence of the amount of PBI–H_3_PO_4_ in the catalytic layer of a high temperature PEMFC, Int J Hydrogen Energ, 35 (2010) 1347–1355.

[20] H. Li, P. Zuo, W. Wu, G. Tang, J. Fang, T. Xu, Z. Yang, Electrode binder design for high-power, low-Pt loading and durable high temperature fuel cells, Energy Environ Sci, 17 (2024) 3651.
